# Early Markers Associated with Crohn’s Disease in Emergency Department Patients with New-Onset Terminal Ileitis: An Exploratory Retrospective Cohort Study

**DOI:** 10.3390/jcm15186947

**Published:** 2026-09-08

**Authors:** Ori Blich, Ruth Lorisso-Nativ, Menachem Schechter, Benjamin Koslowsky, Ofra Carmel, Shlomo Yellinek

**Affiliations:** 1 Department of General Surgery, Eisenberg R&D Authority, Shaare Zedek Medical Centre, The Hebrew University School of Medicine, Jerusalem 9103102, Israel; 2 Hebrew University-Hadassah School of Medicine, The Hebrew University of Jerusalem, Jerusalem 9112002, Israel; 3 Department of Gastroenterology, Shaare Zedek Medical Center, The Hebrew University School of Medicine, Jerusalem 9103102, Israel

**Keywords:** Crohn’s disease, ileitis, risk stratification

## Abstract

**Background and Objectives:** Terminal ileitis is a frequent radiologic finding in patients presenting to the emergency department with acute right lower quadrant pain, yet whether it represents an early manifestation of Crohn’s disease remains challenging to predict. This study aimed to identify early clinical, laboratory, and imaging predictors of Crohn’s disease in patients with newly identified radiologic terminal ileitis to improve early risk stratification. **Materials and Methods:** A retrospective cohort study evaluated adult patients presenting to the emergency department between 2010–2021 with radiologically confirmed terminal ileitis and no prior history of inflammatory bowel disease. Clinical data, laboratory markers, and imaging findings captured at presentation were analyzed. Patients were followed for 12 months for a subsequent Crohn’s disease diagnosis. **Results:** Out of 68 included patients, 23 (33.8%) were diagnosed with Crohn’s disease within one year. Multivariable logistic regression identified lower haemoglobin levels (OR 2.17 per 1 g/dL decrease, 95% CI 1.02–4.76, *p* = 0.044), elevated platelet counts (OR 1.03 per 103/µL, 95% CI 1.01–1.05, *p* = 0.002), and a composite radiologic finding of stenosis or proximal bowel distension (OR 28.5, 95% CI 2.35–345.6, *p* = 0.008) as independent predictors of a subsequent Crohn’s disease diagnosis. Standalone platelet count (AUC = 0.897) and the platelet-to-hemoglobin ratio (AUC = 0.896) demonstrated excellent diagnostic discrimination. **Conclusions:** In this small retrospective cohort, approximately one-third of patients presenting with new-onset radiologic terminal ileitis were subsequently diagnosed with Crohn’s disease within 12 months. Low hemoglobin, elevated platelet count, and radiologic evidence of stenosis or proximal bowel distension were independently associated with this diagnosis. These associations are hypothesis-generating and require external prospective validation before informing clinical decision-making.

## 1. Introduction

Crohn’s disease (CD) is a chronic inflammatory bowel disease characterized by transmural inflammation that may involve any part of the gastrointestinal tract, with the terminal ileum most frequently affected [1,2,3]. Terminal ileitis (TI) is a common radiologic finding, particularly among patients presenting to the emergency department (ED) with acute abdominal pain [1,2,3]. Although CD is a common cause of TI, other aetiologies—including infectious, ischemia, drug-induced, or granulomatous diseases—may present with similar imaging features [4,5].

Due to the broad differential diagnosis of TI, mostly mild and self-limiting, many patients are discharged without further evaluation, and some are later found to have underlying CD. Diagnostic delay in CD is well documented and is associated with an increased risk of strictures, fistulas, and the need for surgery [6,7]. Early identification of patients with TI who are at higher risk of CD could therefore facilitate earlier gastroenterological evaluation and reduce progression and complications.

Mural thickening involved segment length and mesenteric fat stranding have been examined in previous literature; however, their reliability as predictive indicators remains unproven [8]. While hematologic markers like anemia and thrombocytosis are established indicators of inflammation in IBD [9,10], evidence remains limited regarding the integration of clinical, laboratory, and imaging data at ED presentation to predict which patients with TI will ultimately be diagnosed with CD.

We aimed to identify specific ED-based clinical, laboratory, and imaging markers that identify early markers associated with a subsequent CD diagnosis in patients with radiologic terminal ileitis. We hypothesized that synthesizing these accessible data points could enhance early risk stratification and streamline gastroenterology follow-up.

## 2. Methods

### 2.1. Study Design and Setting

This retrospective cohort study was conducted at Shaare Zedek Medical Center, a tertiary 1200-bed, national IBD excellence referral center, in Jerusalem, Israel. Medical records of patients presenting to the emergency department (ED) between 2010 and 2021 were reviewed.

### 2.2. Study Population

The study included adults aged 18–80 years with radiologically confirmed terminal ileitis (TI) on CT or ultrasound, defined as segmental wall thickening or enhancement of the terminal ileum. Patients were required to have at least 12 months of follow-up data. Exclusion criteria were: prior diagnosis of inflammatory bowel disease (IBD), pregnancy, incomplete medical records, or ongoing gastroenterological evaluation for suspected IBD at the time of ED presentation. Study participants were grouped according to the presence or absence of a CD diagnosis within one year of their index ED visit.

### 2.3. Data Collection

Data collected included clinical, laboratory, and imaging variables. Clinical data comprised demographics, presenting symptoms, smoking status, prior abdominal surgery, and emergency department disposition (admission versus discharge). Laboratory data included complete blood count, liver enzymes, albumin, C-reactive protein (CRP), and creatinine. Imaging data included the presence of stenosis, proximal bowel distension, free fluid, collection, and involved segment length. A diagnosis of CD was confirmed based on comprehensive clinical assessment by a board-certified gastroenterologist applying the ECCO diagnostic framework [6]. This assessment integrated available ileocolonoscopic findings (where performed); cross-sectional imaging demonstrating mural thickening, fat stranding, or fibrofatty proliferation; and the overall clinical picture. ECCO diagnostic criteria do not require any single histopathological feature; non-caseating granulomas are present in fewer than 20% of confirmed CD cases [11] and are neither necessary nor sufficient for diagnosis. The diagnosis in each case was based on the totality of available evidence; imaging alone was not accepted as the sole criterion. Qualitative endoscopic features—including aphthous ulcers, deep linear ulcers, cobblestoning, skip lesions, and rectal sparing—were extracted from the attending gastroenterologist’s structured clinical reports through chart review. Systematic use of validated endoscopic scoring systems (SES-CD or CDEIS) was not uniform across the 2010–2021 study period. In the non-CD group, CD was considered effectively excluded in patients who: (a) underwent ileocolonoscopy with normal or non-specific findings; (b) had complete clinical resolution without IBD-specific therapy; and/or (c) had an alternative confirmed diagnosis. Not all non-CD patients had protocolized ileocolonoscopy; outcome misclassification in those without complete endoscopic workup is acknowledged as a limitation. Imaging modality at the index ED visit included CT or ultrasound.

### 2.4. Statistical Analysis

Continuous variables were compared using Student’s *t*-test or Mann–Whitney U test, as appropriate. Categorical variables were analyzed using χ^2^ tests or Fisher’s exact tests. Pearson correlation coefficients were used to assess associations between continuous variables. Variables with *p* < 0.05 in univariate analysis were entered into multivariable logistic regression to identify independent predictors of CD. Diagnostic performance was evaluated using receiver operating characteristic (ROC) curves. Statistical significance was defined as *p* ≤ 0.05 (two-tailed). Optimal diagnostic cutoffs were identified using the Youden Index (maximizing sensitivity + specificity − 1) within the development dataset; this produces optimistic apparent sensitivity and specificity, and all performance metrics should be interpreted as such pending external prospective validation. All candidate predictors reaching *p* < 0.05 in univariate analysis were entered simultaneously into the multivariable model using the forced-entry (ENTER) method to avoid stepwise selection bias in this small sample. Complete-case analysis was applied, with one patient excluded from the multivariable model due to a missing platelet value at presentation. Given the small sample size, the anticipated events-per-variable ratio (EPV) would fall substantially below the recommended minimum of 10; accordingly, all multivariable results should be considered exploratory. Bootstrap internal validation (B = 1000 resamples) was performed to estimate optimism-corrected AUC; calibration slope and intercept are reported in the Supplementary Materials (Table S4; Figure S2).

To assess whether the associations of haemoglobin and platelet count with the outcome were linear, each predictor was additionally modelled using restricted cubic splines (RCS) with three knots placed at the 10th, 50th, and 90th percentiles. Non-linearity was evaluated by a likelihood-ratio test comparing the RCS model to the standard linear specification. LOESS-smoothed probability curves with 95% bootstrap confidence intervals (500 resamples) were generated to visualise the marginal relationship between each predictor and the probability of a subsequent CD diagnosis.

## 3. Results

### 3.1. Study Population and Baseline Characteristics

A total of 68 patients were included in the study. Within 12 months of the index ED visit, 23 patients (33.8%) received a diagnosis of CD, while 45 (66.2%) did not. (Figure S1). Among the 45 non-CD patients, final diagnoses included self-limiting or idiopathic acute ileitis without an identified pathogen (49%), confirmed or suspected infectious ileitis due to *Yersinia enterocolitica*, *Campylobacter* spp., or *Salmonella* spp. (18%), NSAID-associated enteropathy (9%), ischemic ileitis (4%), and other or undetermined aetiology (20%); the full breakdown is provided in Table S1. As detailed in Table 1, there were no significant differences between the CD and non-CD groups regarding age, sex, smoking status, family history of CD, or prior abdominal surgeries. Clinical presentation, including symptoms and vital signs, was comparable between groups and did not significantly predict a subsequent CD diagnosis.

### 3.2. Laboratory Findings

**Figure 1:**
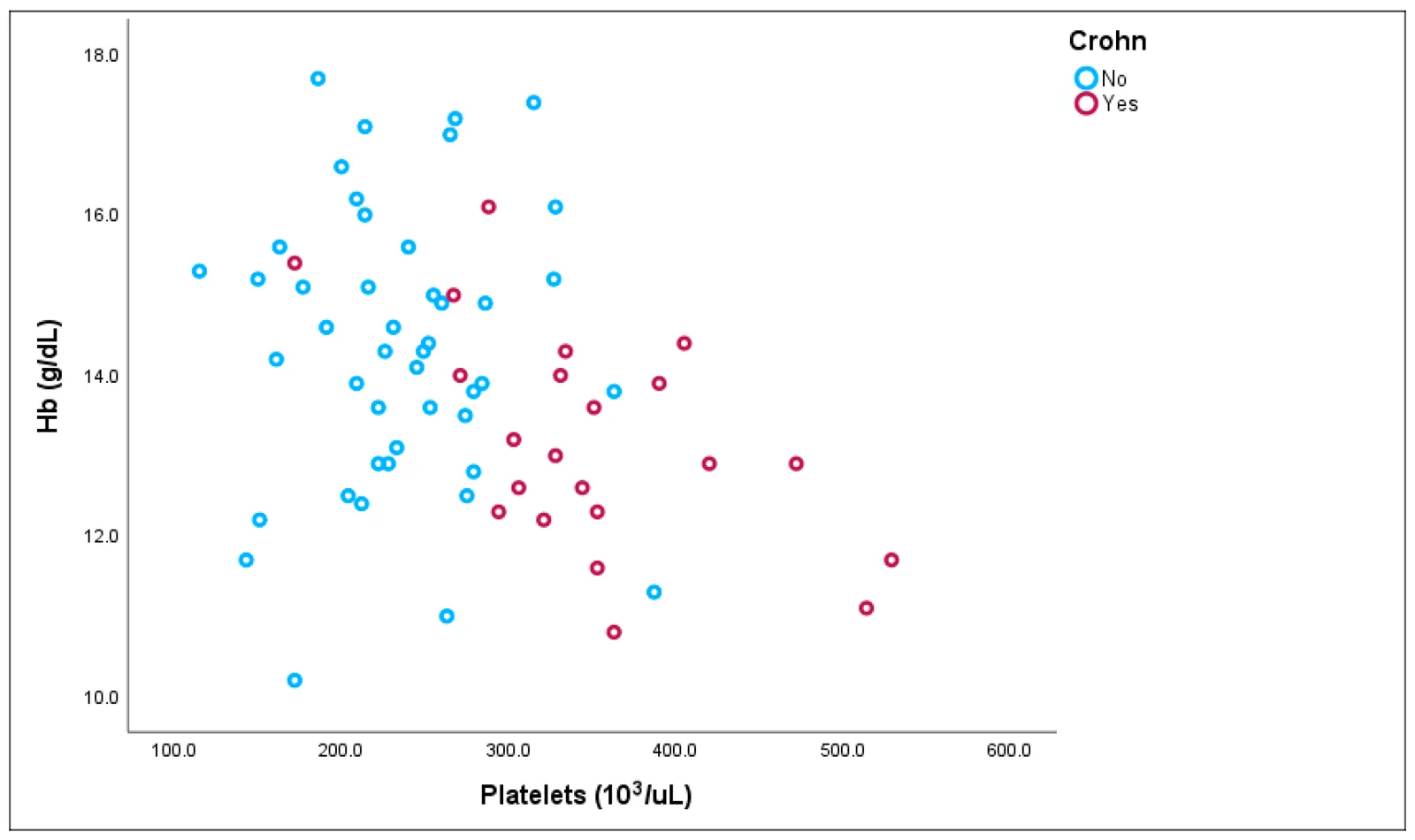
Bi-Variate Scatter Plot Mapping Inter-Relationships Between Hemoglobin Distributions and Absolute Platelet Counts Across Both Cohorts.

Laboratory data are summarized in Table 2. Patients subsequently diagnosed with CD exhibited significantly lower hemoglobin levels (13.18 ± 1.37 vs. 14.34 ± 1.76 g/dL, *p* < 0.01) and higher platelet counts (350.41 ± 82.07 vs. 235.47 ± 57.40 × 10^3^/µL, *p* < 0.001) (Figure 1). The platelet-to-hemoglobin (PLT/Hb) ratio was significantly elevated in the CD group at the time of presentation (27.21 ± 8.24 vs. 16.68 ± 4.75, *p* < 0.001) and in historical records (30.68 ± 16.34 vs. 17.66 ± 5.38, *p* = 0.007). While GGT and ALP were modestly higher in patients with CD (*p* = 0.005, *p* = 0.046), CRP levels at presentation showed no significant intergroup difference.

### 3.3. Predictive Performance of Hematologic Markers

**Figure 2:**
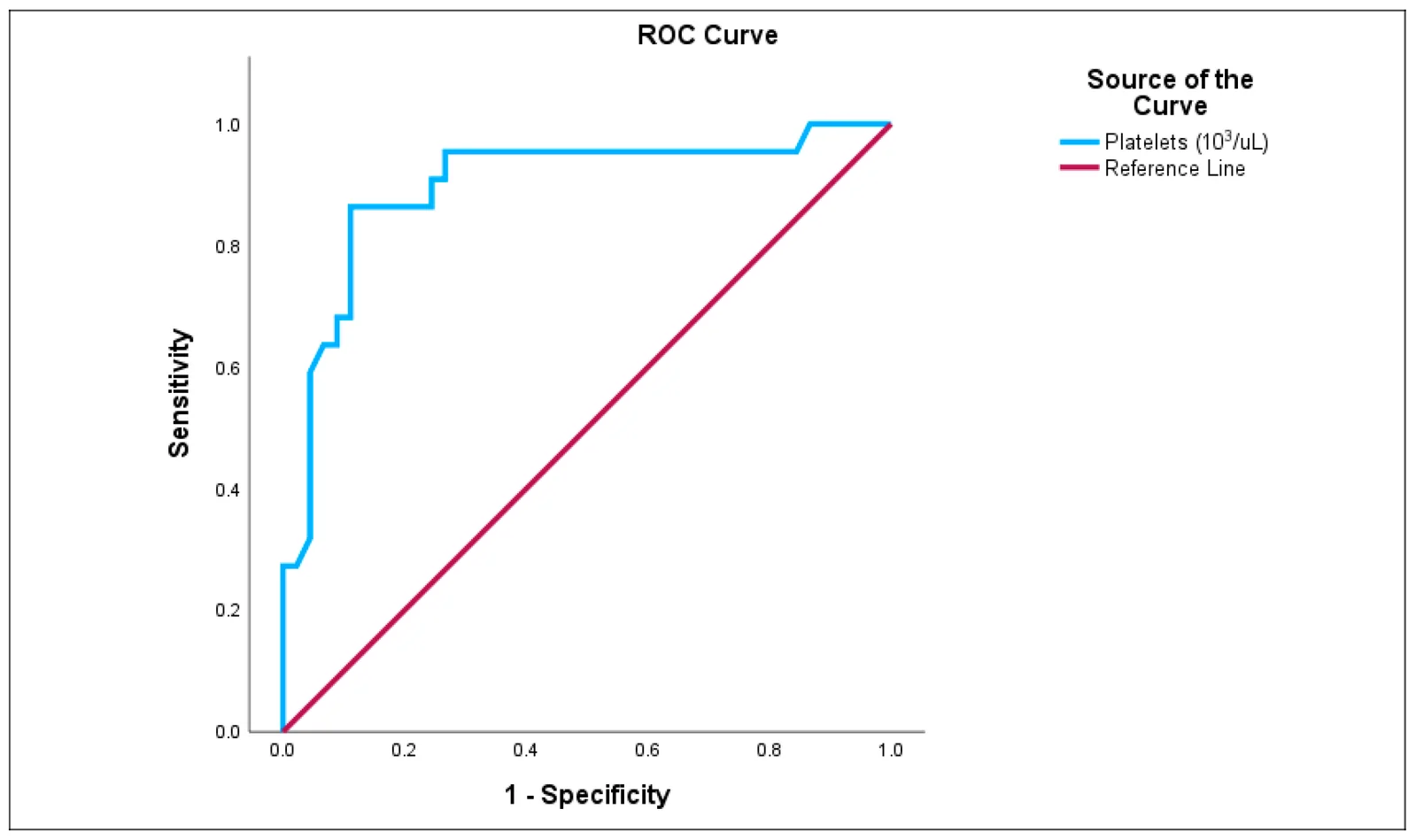
Receiver Operating Characteristic (ROC) Curve for Baseline Platelet Count in Predicting One-Year Crohn’s Disease Progression.

**Figure 3:**
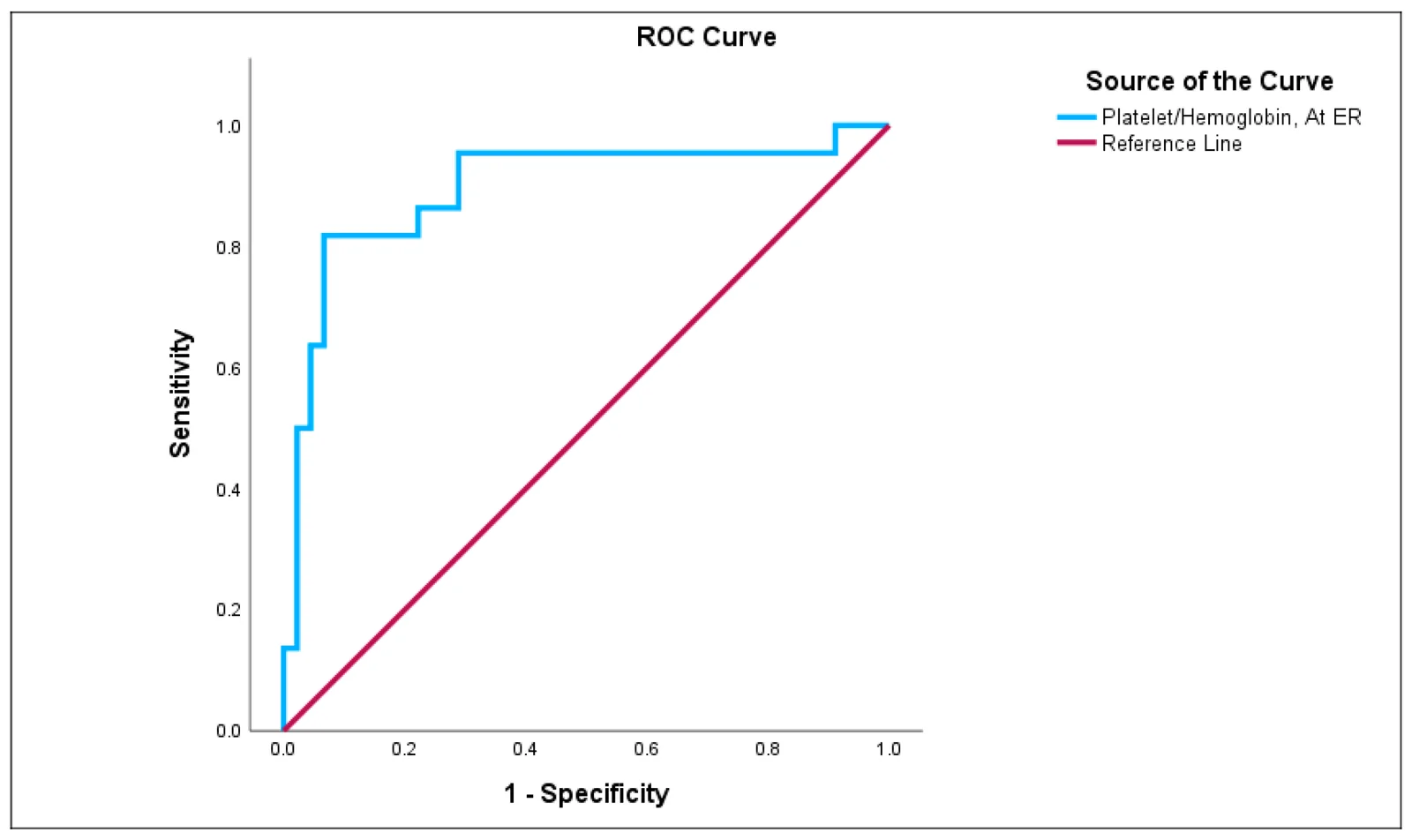
Receiver Operating Characteristic (ROC) Curve for the Platelet-to-Hemoglobin Ratio Parameter Variant Evaluated at Emergency Presentation.

Receiver operating characteristic (ROC) analysis revealed that platelet count had strong discriminative power for CD (AUC = 0.897; 95% CI: 0.809–0.986; *p* < 0.001). An optimal cutoff of 287 × 10^3^/µL yielded a sensitivity of 86.4% (95% CI 65.1–97.1%) and specificity of 88.9% (95% CI 75.9–96.3%) (OR: 50.7, 95% CI: 10.95–234.45) (Figure 2). These performance estimates are derived from the same dataset used to identify the cutoff (Youden Index) and are therefore optimistic; bootstrap-corrected AUC estimates are provided in Supplementary Table S4. The PLT/Hb ratio performed similarly (AUC = 0.896; 95% CI: 0.803–0.989; *p* < 0.001). A threshold of 22.48 provided 81.8% sensitivity (95% CI 59.7–94.8%) and 93.3% specificity (95% CI 81.7–98.6%) (OR: 63.0, 95% CI: 12.8–310.7) (Figure 3). All performance estimates should be considered internally validated exploratory findings pending prospective external validation.

### 3.4. Imaging Characteristics

Patients with CD were significantly more likely to present with stenosis (42.1% vs. 13.2%, *p* = 0.021), proximal bowel distension (33.3% vs. 10.3%, *p* = 0.039) and free fluid/intra-abdominal collection (72.7% vs. 43.9%, *p* = 0.029). A composite variable of stenosis or distension was highly associated with CD; 50% of patients with either finding received a diagnosis of CD, compared to only 15.4% of those without (*p* = 0.004) (Table 3).

### 3.5. Multivariable Analysis

Multivariable logistic regression identified three independent predictors of a subsequent CD diagnosis: lower haemoglobin levels (OR 2.17 per 1 g/dL decrease, 95% CI: 1.02–4.76, *p* = 0.044), higher platelet count (OR 1.03 per 103/µL, 95% CI: 1.01–1.048, *p* = 0.002) and stenosis or proximal bowel distension (OR 28.51, 95% CI: 2.352–345.581, *p* = 0.008) (Table 4). Male sex was also identified as a significant adjusted predictor (OR 21.84); however, this estimate is likely to reflect sparse-data bias rather than a true biological effect, as confirmed by Firth-penalised logistic regression (Supplementary Table S3), where the OR is attenuated to 4.12 (95% CI 0.73–31.6, *p* = 0.11, non-significant). Male sex is not presented as a reliable independent predictor. The wide confidence intervals throughout Table 4 reflect the small sample size and EPV of 4.4.

To evaluate the functional form of the associations, haemoglobin and platelet count were additionally modelled using restricted cubic splines (RCSs). Neither predictor showed a statistically significant departure from linearity (haemoglobin: *p* = 0.73; platelet count: *p* = 0.56), supporting the use of standard logistic regression. LOESS-smoothed probability curves and RCS-based adjusted odds ratio plots are presented in Supplementary Figure S3.

### 3.6. Clinical Suspicion and Decision-Making

Exploratory analysis of clinical documentation suggested that physician suspicion aligned with eventual outcomes. Patients later diagnosed with CD were significantly more likely to be admitted to the hospital (56.7% vs. 15.8%, *p* < 0.005) and receive specific recommendations for gastroenterological follow-up (43.9% vs. 8.3%, *p* = 0.038) and colonoscopy (45.9% vs. 12.5%, *p* = 0.020). These subjective variables were excluded from the predictive model. Imaging modality was also significantly associated with CD outcome: CT was performed in 57 of 68 patients (83.8%) and ultrasound in 11 of 68 (16.2%). All 23 patients subsequently diagnosed with CD had CT imaging; none had ultrasound-only evaluation (Fisher’s exact *p* = 0.010).

## 4. Discussion

In this retrospective cohort of adults presenting to the emergency department with radiologic terminal ileitis (TI), approximately one-third were subsequently diagnosed with CD, a rate consistent with previously reported literature [4,5]. This finding underscores the prognostic importance of TI beyond mere radiologic observation and emphasizes the need for careful early evaluation in this setting, especially given that the epidemiological burden of inflammatory bowel diseases in Israel reflects a substantial and rising clinical challenge [3]. Distinguishing between symptomatic non-Crohn’s ileitis and true CD remains a long-standing clinical dilemma due to their highly overlapping clinical, endoscopic, and histopathological findings [12].

Early identification of CD has vital clinical implications. Timely diagnosis allows intervention during a critical “window of opportunity,” when early anti-inflammatory treatment may alter disease progression, reduce rates of stricture formation or penetrating complications, and prevent cumulative bowel damage [13]. Several studies have emphasized the importance of identifying clinical or laboratory “red flags” to prompt earlier diagnostic evaluation for inflammatory bowel disease [14]. Our study identified several independent predictors associated with a subsequent diagnosis of CD, including thrombocytosis, lower hemoglobin levels, and radiologic evidence of stenosis or proximal bowel distension.

Importantly, most baseline demographic characteristics, presenting symptoms, and vital signs, including body temperature, mean blood pressure, and heart rate, were similar between groups. This lack of clinical variance underscores the difficulty of distinguishing CD from other acute etiologies of terminal ileitis based on clinical presentation alone, a problem mirrored in broader emergency settings. Previous studies have highlighted that while acute ileitis on imaging often represents the first manifestation of inflammatory bowel disease, its clinical presentation is highly confounded by secondary infectious, drug-induced, or ischemic etiologies. Consequently, the absence of distinct initial acute symptoms frequently creates uncertainty regarding which patients require close, long-term follow-up. Furthermore, it has been shown that while acute-onset abdominal pain, weight loss, or diarrhea often raise clinical suspicion for CD in hospitalized cohorts, these presenting symptoms do not consistently or reliably correlate with the final diagnosis, mandating the development of objective, multi-variable risk-stratification models [15,16].

The associations between thrombocytosis, anemia and active inflammation are well recognized in inflammatory bowel disease and likely reflect systemic inflammatory activity [9,10]. Platelet activation contributes to the inflammatory cascade and has been consistently reported in active IBD, aligning with previous findings of elevated platelet counts in CD. The platelet-to-hemoglobin ratio (PLT/Hb), which integrates two inflammation-related parameters, demonstrated good diagnostic discrimination in our cohort (AUC = 0.896). These markers may serve as simple and readily available adjuncts when interpreting nonspecific inflammatory markers in patients presenting with terminal ileitis, particularly when initial acute-phase reactants show no significant difference between groups at presentation.

Interestingly, our data also revealed that long-term hematologic trends, such as historical platelet counts within the prior 12 months, remained significantly higher in the eventual CD cohort. Such elevations in platelet count are recognised to reflect the pre-symptomatic stage of CD and IBD, where early inflammatory changes in the gut and in systemic markers precede the onset of clinical symptoms [9,10]. Similarly, faecal calprotectin levels have been shown to be elevated weeks to months before clinical symptom onset in patients subsequently diagnosed with IBD, further supporting the concept of an early inflammatory window that may be detectable at the time of an initial ED presentation.

Radiological predictors—particularly stenosis and proximal bowel distension—were strongly associated with CD in our study. These findings likely reflect early fibro-stenotic changes characteristic of transmural CD, consistent with the recognized inflammation–fibrosis sequence. When these structural changes compound into clear clinical stenosis or distension, the likelihood of a subsequent CD diagnosis rises dramatically, as demonstrated by our composite finding where 50% of patients with either feature progressed to CD compared to only 15.4% without them (*p* = 0.004). While prior studies have noted that general findings such as ileocecal/mural thickening, fat stranding, and involved segment length are common diagnostic markers, their reliability as solitary predictors can be variable [8]. Our results demonstrate that cross-sectional combination of macro-structural radiologic complications and sensitive hematologic biomarkers can lead to future early risk stratification. This aligns with data from previous studies evaluating isolated terminal ileitis to determine when it represents an incidental, self-limiting finding versus an early manifestation of CD, emphasizing that structural and luminal evaluation is key to preventing diagnostic delay [17].

Clinical documentation variables, although inherently subjective, also revealed meaningful patterns. Patients who were hospitalized or were recommended to complete gastroenterological investigation or colonoscopy were significantly more likely to receive a later diagnosis of CD. While these findings likely reflect clinician suspicion rather than objective disease markers, they emphasize the value of clinical judgment in the early evaluation of equivocal TI. This clinical intuition aligns well with international practice standards, which advocate objective endoscopic and histological verification to guide appropriate medical optimization [6]. Taken together, these findings suggest that patients presenting with TI who exhibit thrombocytosis, anemia, or stenosis or proximal bowel distension on imaging may benefit from early gastroenterology referral and timely colonoscopy [6]. For emergency physicians, the presence of these features should raise suspicion for underlying CD and may prompt early specialist evaluation. Integrating these simple, widely available parameters into ED decision-making may facilitate earlier identification of patients likely to be subsequently diagnosed with inflammatory bowel disease, enabling timely management before severe, irreversible structural bowel damage takes place.

Although these findings require validation in larger prospective cohorts, they highlight the potential role of combining simple laboratory and radiologic parameters to optimize the early identification of CD among patients presenting with terminal ileitis. Given the exploratory nature of this single-center retrospective study with EPV < 5, the associations reported here are hypothesis-generating only and must not be used for clinical decision-making pending external prospective validation. Future prospective studies should include systematic imaging with CT, routine measurement of CBC-derived indices including RDW and MPV, standardized endoscopic scoring (SES-CD), and protocolized 12-month follow-up for all patients with new-onset radiologic terminal ileitis regardless of initial severity.

This study is primarily limited by its retrospective, single-center design at a large tertiary IBD referral center. Importantly, while the center functions as a national IBD referral site, this designation applies to the gastroenterology and surgical outpatient clinics; the ED serves a local catchment-area population not selected by subspecialty. The events-per-variable ratio (EPV = 4.4; 22 CD events, 5 predictors) falls substantially below the recommended minimum of 10, substantially increasing the risk of overfitting and unreliable coefficient estimates; all multivariable results must be considered exploratory. An important limitation specific to the Israeli healthcare context is the structure of health maintenance organizations (Kupot Holim; KH). Patients discharged from our ED who underwent colonoscopy and received a CD diagnosis through their KH without returning to our institution would not be captured in our follow-up records and would be misclassified as non-CD. This likely results in underestimation of the true CD incidence. Imaging modality represents a source of ascertainment bias: CT was performed in 57 of 68 patients (83.8%) and ultrasound in 11 of 68 (16.2%), and all 23 CD diagnoses were made in CT-imaged patients. The lower sensitivity of ultrasound for detecting stenosis, distension, and fat stranding may have contributed to the complete absence of CD diagnoses in the ultrasound subgroup. The retrospective design precluded uniform use of endoscopic scoring systems (SES-CD/CDEIS); specific endoscopic and histopathological findings were extracted from clinical reports rather than structured data fields. Outcome review was not blinded to ED presentation data. RDW, MPV, and PDW were not systematically recorded across the 2010–2021 study period, limiting the completeness of the inflammatory marker profile. Calendar year, NSAID and corticosteroid use, iron supplementation status, and access to gastroenterology care were not systematically recorded as potential confounders. Missing data was handled by complete-case analysis; laboratory data were predominantly unavailable in patients discharged directly from the ED, who were also less likely to receive a CD diagnosis, introducing informative missingness that may bias results toward stronger associations. Patients without a confirmed CD diagnosis within the 12-month follow-up window were categorized as non-CD; cases of delayed presentation, incomplete diagnostic workup, or diagnosis occurring outside our institution may have been missed. We are now in the process of a follow-up large cohort study in collaboration with one of the largest KHs in Israel and hope to publish our results in the near future.

## 5. Conclusions

This exploratory retrospective cohort study demonstrates that approximately one-third of patients presenting to the emergency department with new-onset radiologic terminal ileitis are subsequently diagnosed with CD within one year. Low hemoglobin, elevated platelet count, and radiologic evidence of stenosis or proximal bowel distension were independently associated with this outcome in multivariable logistic regression. These associations are hypothesis-generating and must not be used for clinical decision-making pending external prospective validation in larger, multicenter cohorts with standardized follow-up pathways.

## Figures and Tables

**Table 1:** Baseline Demographics, Clinical Presentation, and Vital Signs of the Patient Cohort.

Variable	Crohn’s Disease	Non-Crohn’s	*p*-Value
	(*n* = 23)	Disease (*n* = 45)	
Male sex, % (*n*)	65.2% (15/23)	57.8% (26/45)	0.553
Age at presentation,	29 (18–74)	29 (18–79)	0.640
median (IQR), years			
Smoking status, % (*n*)	12.5% (1/8)	19.0% (4/21)	1.000
BMI, median (range)	26 (17–38) (*n* = 6)	25 (16.5–36.3)	0.866
		(*n* = 26)	
History of abdominal	23.1% (3/13)	22.2% (6/27)	0.697
surgery, % (*n*)			
Familial Crohn’s	27.3% (3/11)	26.7% (4/15)	1.000
disease, % (*n*)			
Right lower quadrant	50.0% (10/20)	72.1% (31/43)	0.087
pain, % (*n*)			
Prior similar episodes,	91.7% (11/12)	56.3% (9/16)	0.088
% (*n*)			
Arthritis, % (*n*)	33.3% (2/6)	36.4% (4/11)	1.000
Weight loss, % (*n*)	10.0% (2/20)	2.3% (1/43)	0.234
Body temperature (°C), median [range]	36.5 (35.6–38.3)	36.8 (35.7–39.4)	0.368
Mean blood pressure	92.2 (40.7–110.0)	91.0 (62.0–143.0)	0.989
(mmHg), median			
[range]			
Heart rate (BPM),	87 (69–130)	85.5 (54–141)	0.426
median [range]			
RLQ tenderness on	81.8% (18/22)	84.4% (38/45)	1.000
examination, % (*n*)			
Local peritonitis on	18.2% (4/22)	15.6% (7/45)	1.000
examination, % (*n*)			

BMI, body mass index; BPM, beats per minute; IQR, interquartile range; RLQ, right lower quadrant.

**Table 2:** Acute Admission and 12-Month Historical Laboratory Findings.

Variable	Non-Crohn’s Disease (Mean ± SD)	Crohn’s Disease (Mean ± SD)	*p*-Value
WBC (10^3^/µL)	10.37 ± 3.50 (*n* = 45)	10.65 ± 3.84 (*n* = 22)	0.754
Neutrophils (%)	72.43 ± 9.67 (*n* = 27)	72.58 ± 14.23 (*n* = 13)	0.583
Lymphocytes (%)	17.94 ± 7.91 (*n* = 27)	17.78 ± 12.67 (*n* = 13)	0.436
Hemoglobin (g/dL)	14.34 ± 1.76 (*n* = 45)	13.18 ± 1.37 (*n* = 22)	* 0.009
Platelets (10^3^/µL)	235.47 ± 57.40 (*n* = 45)	350.41 ± 82.07 (*n* = 22)	* 0.001
Creatinine (mg/dL)	0.85 ± 0.20 (*n* = 45)	0.77 ± 0.13 (*n* = 22)	0.135
Alkaline Phosphatase	72.39 ± 24.05 (*n* = 44)	91.55 ± 52.09 (*n* = 22)	* 0.046
(U/L)			
AST (U/L)	24.66 ± 5.38 (*n* = 44)	24.64 ± 15.07 (*n* = 22)	* 0.005
ALT (U/L)	23.16 ± 10.86 (*n* = 44)	21.48 ± 15.49 (*n* = 21)	0.121
GGT (U/L)	20.70 ± 9.50 (*n* = 44)	34.32 ± 24.95 (*n* = 22)	* 0.005
CRP at Presentation	6.05 ± 8.03 (*n* = 22)	7.45 ± 7.50 (*n* = 15)	0.286
(mg/L)			
Albumin at	3.30 ± 1.56 (*n* = 6)	3.46 ± 0.70 (*n* = 5)	0.358
Presentation (g/dL)			
Fecal Calprotectin	161.50 ± 400.13 (*n* = 8)	808.60 ± 1195.93	0.079
(µg/g)		(*n* = 5)	
Platelet/Hemoglobin	16.68 ± 4.75 (*n* = 45)	27.21 ± 8.24 (*n* = 22)	* 0.001
Ratio			
Historical			
Laboratory Trends			
CRP (mg/L)	9.46 ± 13.84 (*n* = 8)	12.04 ± 9.46 (*n* = 4)	0.396
Hemoglobin (g/dL)	14.06 ± 1.42 (*n* = 28)	13.50 ± 1.36 (*n* = 6)	0.366
Platelets (10^3^/µL)	243.64 ± 59.01 (*n* = 28)	404.17 ± 190.13 (*n* = 6)	* 0.004
Albumin (g/dL)	4.29 ± 0.35 (*n* = 11)	4.12 ± 0.50 (*n* = 4)	0.553
Serum Iron (µg/dL)	82.84 ± 27.41 (*n* = 19)	60.50 ± 30.71 (*n* = 4)	0.223
Ferritin (µg/L)	63.66 ± 34.89 (*n* = 10)	172.84 ± 277.43 (*n* = 5)	0.903
Vitamin B12 (pg/mL)	439.06 ± 219.91	441.20 ± 75.69 (*n* = 5)	0.551
	(*n* = 18)		
Platelet/Hemoglobin	17.66 ± 5.38 (*n* = 28)	30.68 ± 16.34 (*n* = 6)	* 0.007
Ratio			

* *p* < 0.05; ALT, alanine aminotransferase; AST, aspartate aminotransferase; CRP, C-reactive protein; GGT, gamma-glutamyl transferase; WBC, white blood cell count.

**Table 3:** Imaging Characteristics at Initial Emergency Presentation.

Imaging Feature	Crohn’s Disease	Non-Crohn’s	*p*-Value
	(*n* = 23)	Disease (*n=* 45)	
Stenosis, % (*n*)	42.1% (8/19)	13.2% (5/38)	* 0.021
Proximal bowel	33.3% (7/21)	10.3% (4/39)	* 0.039
distension, % (*n*)			
Composite: stenosis or	50.0% (11/22)	15.4% (6/39)	* 0.004
distension, % (*n*)			
Fistulation, % (*n*)	9.1% (2/22)	0.0% (0/41)	0.118
Colon/cecum	14.3% (3/21)	26.8% (11/41)	0.346
involvement, % (*n*)			
Free fluid and/or fluid	72.7% (16/22)	43.9% (18/41)	* 0.029
collection, % (*n*)			

* *p* < 0.05.

**Table 4:** Independent Predictors of Crohn’s Disease via Multivariable Logistic Regression.

Variable	Unadjusted OR	Univariate *p*-Value	Adjusted OR	Multivariable 95% CI	Multivariable *p*-Value
Stenosis or distension	5.5	* 0.004	28.51	2.352–345.581	* 0.008
Platelet count (per 10^3^/µL)	—–	* 0.001	1.03	1.011–1.048	* 0.002
Haemoglobin (g/dL), per 1 g/dL decrease	—–	* 0.009	2.17	1.02–4.76	* 0.044
Sex (Male)	1.37	0.553	21.84	1.762–270.786	* 0.016
Age at presentation	—–	0.640	0.99	0.936–1.045	0.694

* *p* < 0.05. The adjusted ORs for male sex (OR 21.84) and stenosis/distension (OR 28.51) should be interpreted with caution due to sparse-data bias and near-separation in this small dataset. Firth-penalised logistic regression (Supplementary Table S3) substantially attenuates both estimates; male sex is no longer statistically significant under that specification.

## Data Availability

The original contributions presented in this study are included in the article/Supplementary Material. Further inquiries can be directed to the corresponding author.

## Supplementary Materials

The following supporting information can be downloaded at: https://www.mdpi.com/article/10.3390/jcm15186947/s1, Figure S1. STROBE Patient Flow Diagram. Supplementary Table S1. Missing Data, Non-CD Diagnoses, and Re-presentations, Part A—Variable availability by group. Part B—Final diagnoses in non-CD group (*n* = 45). Part C—Non-CD follow-up investigations during 12-month period. Supplementary Table S2. NLR/PLR Analysis—40-Patient Subgroup (WBC Differential Available); Supplementary Table S3. Firth-Penalised Logistic Regression (*n* = 66, 22 CD events, EPV = 4.4). Supplementary Table S4. Bootstrap Internal Validation (B = 1000 Resamples). Supplementary Table S5. PROBAST Risk-of-Bias Assessment. Figure S2. Calibration Plot—Predicted vs. Observed Crohn’s Disease Probability. Figure S3. LOESS-smoothed probability curves and restricted cubic spline (RCS) adjusted odds ratio plots for haemoglobin and platelet count.
