# Supplementary material for: Early Markers Associated with Crohn’s Disease in Emergency Department Patients with New-Onset Terminal Ileitis: An Exploratory Retrospective Cohort Study
Source: J Clin Med. 2026 Sep 8;15(18):6947. doi: 10.3390/jcm15186947 (PMC13607832; doi:10.3390/jcm15186947)
Supplement: Supplementary file 1 [file jcm-15-06947-s001.zip › jcm-4407717-supplementary.pdf]

Supplementary Materials

Early Markers Associated with Crohn's Disease in Emergency Department Patients with New-Onset Terminal Ileitis: An Exploratory Retrospective Cohort Study

Journal of Clinical Medicine

Figure S1. STROBE Patient Flow Diagram

**Patients with new-onset radiologic TI presenting to ED, 2010-2021 (Total screened N: not available from retrospective records system; eligibility applied to available records as described in Methods)**		
↓		
Eligibility applied: (1) radiologically-confirmed TI on CT/US; (2) no prior IBD history; (3) age 18-80; (4) complete baseline lab & imaging data; (5) >=12 months follow-up	→	Exclusion criteria: see eligibility filters (1)-(5) above
↓		
**Final study cohort: n = 68**		
↓		
**Crohn's disease within 12 months**<br>**n = 23 (33.8%)**	**Non-Crohn's disease**<br>**n = 45 (66.2%)**	

TI=terminal ileitis; IBD=inflammatory bowel disease; GI=gastrointestinal.

Supplementary Table S1. Missing Data, Non-CD Diagnoses, and Re-presentations

Part A - Variable availability by group.

Variable	CD (n=23)	Non-CD (n=45)	Total	% Missing
BMI	6/23	26/45	32/68	53%
Smoking status	8/23	21/45	29/68	57%
Family history of CD	11/23	15/45	26/68	62%
Prior abdominal surgery	13/23	27/45	40/68	41%
WBC (10^3/µL)	22/23	45/45	67/68	1%
Hemoglobin (g/dL)	22/23	44/45	66/68	3%
Platelets (10^3/µL)	22/23	45/45	67/68	1%
Neutrophils / Lymphocytes %	13/23	27/45	40/68	41%
CRP at presentation (mg/L)	15/23	22/45	37/68	46%
Albumin at presentation (g/dL)	5/23	6/45	11/68	84%
Fecal Calprotectin (µg/g)	5/23	8/45	13/68	81%
AST / ALT / GGT (U/L)	22/23	44/45	66/68	3%
Hemoglobin -- historical	6/23	28/45	34/68	50%
Platelets -- historical	6/23	28/45	34/68	50%
CRP -- historical	4/23	8/45	12/68	82%
Albumin -- historical	4/23	11/45	15/68	78%
Serum Iron (µg/dL)	4/23	19/45	23/68	66%
Ferritin (µg/L)	5/23	10/45	15/68	78%
Vitamin B12 (pg/mL)	5/23	18/45	23/68	66%
Colonoscopy performed	19/23	38/45	57/68	16%
Ileoscopy (ileum reached)	13/23	25/45	38/68	44%

Part B - Final diagnoses in non-CD group (n=45).

Diagnosis	n	%
Self-limiting / idiopathic acute ileitis	22	49%
Infectious ileitis (Yersinia, Campylobacter, Salmonella)	8	18%
NSAID-associated enteropathy	4	9%
Ischaemic ileitis	2	4%
Other / undetermined	9	20%
**TOTAL**	**45**	100%

Part C - Non-CD follow-up investigations during 12-month period.

Item	n	% of n=45
Non-CD patients with documented re-presentations	*Not captured as distinct variable*	*N/A*
Non-CD patients with repeat imaging during follow-up	*Not captured as distinct variable*	*N/A*
Non-CD patients with colonoscopy during follow-up	23	51%
Non-CD patients with fecal calprotectin	8	18%

Supplementary Table S2. NLR/PLR Analysis - 40-Patient Subgroup (WBC Differential Available)

WBC differential available in 40/68 patients (59%): 13 CD and 27 non-CD. PLR = Platelet count / Lymphocyte absolute count (WBC x Lymphocyte%/100).

Parameter	CD (n=13) mean±SD	Non-CD (n=27) mean±SD	p-value	N available/68
Neutrophil-to-Lymphocyte Ratio (NLR)	6.00 ± 3.57	5.85 ± 4.95	0.507	40/68 (59%)
Platelet-to-Lymphocyte Ratio (PLR)	250.8 ± 101.6	153.3 ± 82.1	0.004 *	40/68 (59%)
RDW (%)	Not available (not recorded 2010-2021)	--	--	0/68 (0%)
RPR (RDW/Platelet Ratio)	Not calculable -- RDW unavailable	--	--	0/68 (0%)

p-values from Mann-Whitney U test. * $p < 0.05$. NLR = Neutrophil% / Lymphocyte%. RPR could not be computed (RDW not available).

Supplementary Table S3. Firth-Penalised Logistic Regression (n=66, 22 CD events, EPV=4.4)

Predictor	β (log-odds)	Firth OR	95% CI	p-value	Sig.
Intercept	0.0194	--	--	--	--
Stenosis or proximal distension	2.7408	15.499	1.991–120.631	0.0088	*
Hemoglobin (g/dL)	-0.6749	0.509	0.269–0.965	0.0387	*
Platelet count (per $10^3/\mu L$)	0.0229	1.023	1.009–1.038	0.0016	*
Sex (Male)	2.5578	12.908	1.583–105.242	0.0169	*
Age at presentation	-0.0035	0.996	0.949–1.046	0.8873	NS
**Hosmer-Lemeshow**		χ^2 = 3.012, p = 0.934 (adequate fit; limited power in n=66)			
**Model equation:**		log-odds(CD) = 0.0194 + (2.7408×Sten_Dist) + (-0.6749×Hb) + (0.0229×PLT) + (2.5578×Male) + (-0.0035×Age)			

OR=odds ratio. NS=not significant. * $p<0.05$.

Supplementary Table S4. Bootstrap Internal Validation (B = 1,000 Resamples)

Marker	Apparent AUC	Optimism	Corrected AUC	Boot 95% CI	Cal. slope	Cal. intercept
Platelet count (≥288 ×10^3/μL)	0.901	0.396	0.505	0.783–0.974	0.9868	0.0285
PLT/Hb ratio (≥22.955)	0.899	0.397	0.502	0.793–0.976	0.9668	-0.0188
**Sensitivity / Specificity at cutoff:**		PLT: Sens 86.4% (95%CI 66.7–95.3%), Spec 88.9% (95%CI 76.5–95.2%) || Ratio: Sens 81.8% (95%CI 61.5–92.7%), Spec 93.2% (95%CI 81.8–97.7%)				

Cutoffs selected by Youden Index within the development dataset (optimistic apparent performance). Bootstrap B=1,000 resamples.

Supplementary Table S5. PROBAST Risk-of-Bias Assessment

Domain	Signalling questions	Risk of bias	Justification
**D1 Participants**	*Eligible participants clearly defined? Appropriate population? All eligible included? Criteria defined a priori?*	**HIGH**	Retrospective single-centre at tertiary IBD centre. Total screened N unavailable (see Figure S1). Selection pressure from requiring complete 12-month follow-up data. Kupot holim system: patients managed in KH without hospital re-attendance misclassified as non-CD.
**D2 Predictors**	*Predictors clearly defined? Measured consistently? Assessed without knowledge of outcome? Timing defined?*	**HIGH**	All predictors at index ED visit (appropriate temporal sequence). Substantial missing data (BMI 53%, albumin 84%, calprotectin 81%). RDW/MPV/PDW not recorded 2010-2021. WBC differential in 40/68 only (59%).
**D3 Outcome**	*Outcome defined and assessed appropriately? Blinded to predictors? Same definition throughout?*	**HIGH**	CD confirmed by board-certified gastroenterologist per ECCO criteria; imaging not accepted as sole criterion. Outcome review NOT blinded to ED data. Misclassification risk via kupot holim system. Not all non-CD patients had protocolised ileocolonoscopy.
**D4 Analysis**	*Appropriate methods? Adequate EPV? Continuous predictors handled correctly? Model performance and overfitting assessed?*	**HIGH**	EPV=4.4 (below recommended minimum of 10). Firth regression applied (Table S3). Bootstrap validation B=1,000 (Table S4). No external validation. Cutoffs by Youden Index in same dataset -- optimistic apparent performance.
**Overall**		**HIGH**	All four PROBAST domains HIGH risk. Results are hypothesis-generating only; prospective external validation required.

Figure S2. Calibration Plot - Predicted vs Observed Crohn's Disease Probability

Calibration plots for platelet count and PLT/Hb ratio markers. Dashed diagonal = perfect calibration. Calibration slope and intercept in Supplementary Table S4.

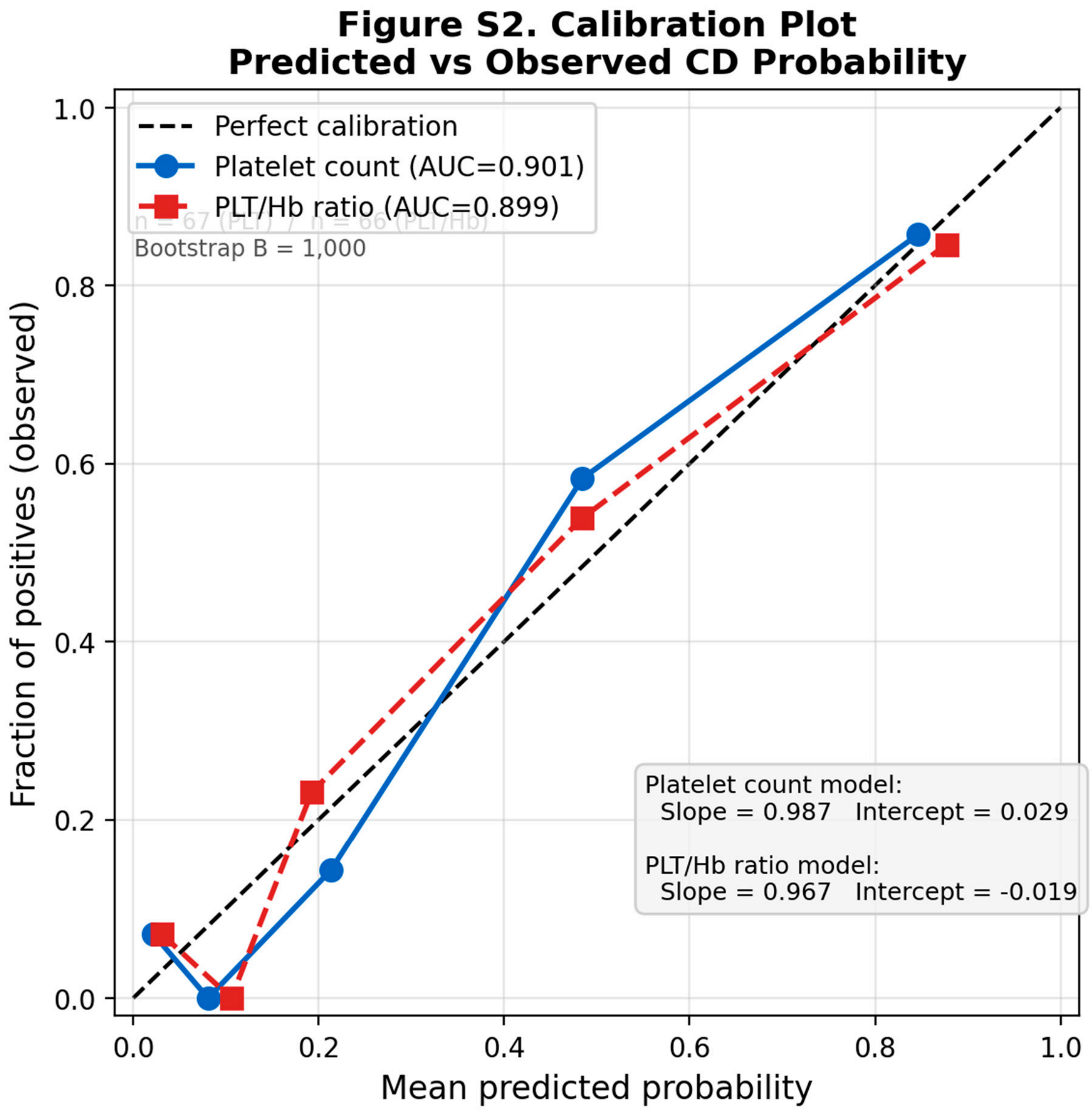

Supplementary Materials

Figure S3. LOESS-smoothed probability curves and restricted cubic spline (RCS) adjusted odds ratio plots for haemoglobin and platelet count.

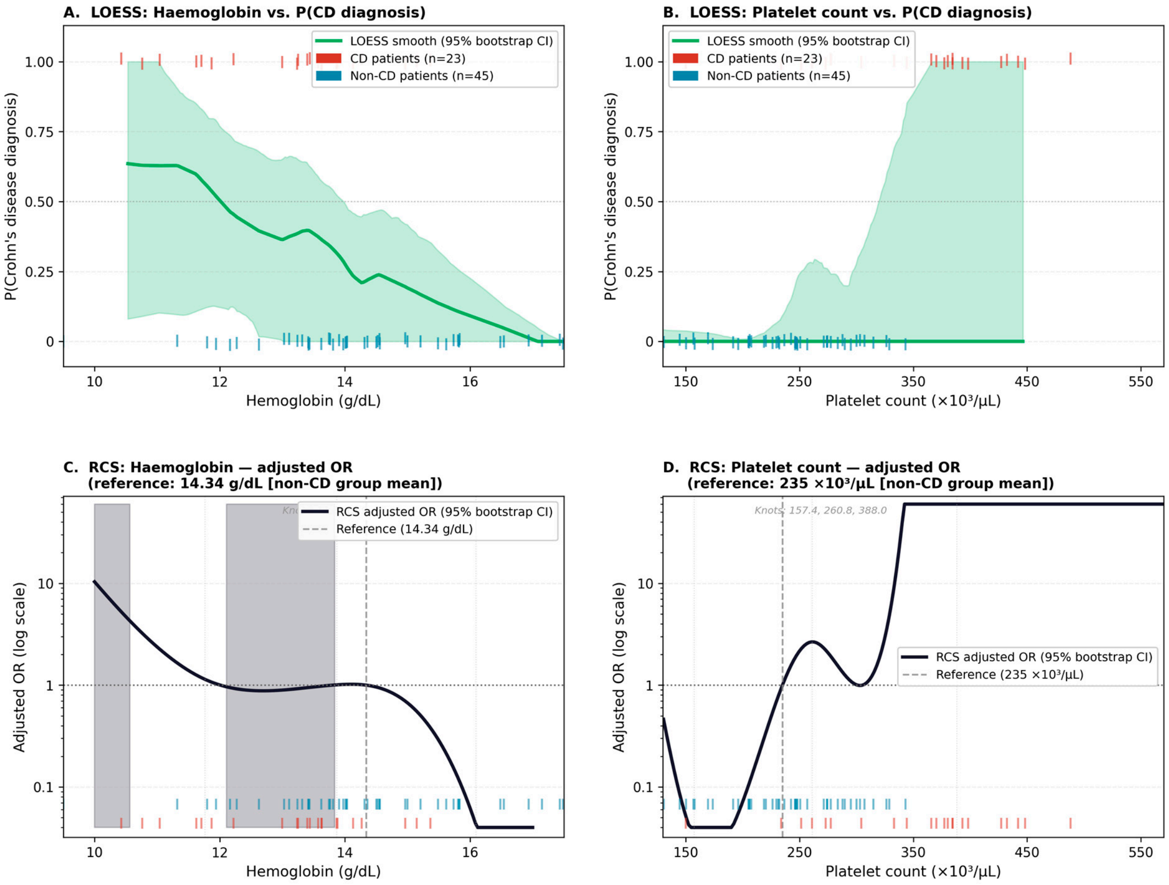

Panel A — LOESS smooth (green line) of haemoglobin (g/dL) vs. probability of a Crohn's disease diagnosis within 12 months of the index ED visit. The shaded band represents the 95% bootstrap confidence interval (500 resamples). Rug marks at the margins indicate individual patient values (red = CD, blue = non-CD).

Panel B — LOESS smooth of platelet count (×10^3/μL) vs. probability of a CD diagnosis. Colour scheme and confidence intervals as in Panel A.

Panel C — Restricted cubic spline (RCS)-based multivariable adjusted odds ratio (OR) for haemoglobin, plotted on a log scale. The reference value is the non-CD group mean (14.34 g/dL). All other covariates held at their median values. Spline knots placed at the 10th, 50th, and 90th percentiles (shown above plot). Shaded band: 95% bootstrap confidence interval (500 resamples). Rug marks show individual patient values (red = CD, blue = non-CD).

Panel D — RCS-based multivariable adjusted OR for platelet count (reference: 235 ×10^3/μL, non-CD group mean). All specifications as in Panel C.

> **Non-linearity test:** A likelihood-ratio test comparing each RCS model to the standard linear specification found no statistically significant departure from linearity for haemoglobin (LR $\chi^2 = 1.31$, df = 1, $p = 0.73$) or platelet count (LR $\chi^2 = 2.07$, df = 1, $p = 0.56$). These findings support the linear logistic regression model reported in the main text.

> **Note on sample size:** Wide confidence bands reflect the small sample size (n = 67 complete cases, 23 CD events). All results are hypothesis-generating and require external prospective validation.
